# Supplementary material for: Mechanistic insight into bacterial entrapment by septin cage reconstitution
Source: Nat Commun. 2021 Jul 23;12:4511. doi: 10.1038/s41467-021-24721-5 (PMC8302635; doi:10.1038/s41467-021-24721-5)
Supplement: Supplementary file 1 — Supplementary Information [file 41467_2021_24721_MOESM1_ESM.pdf]

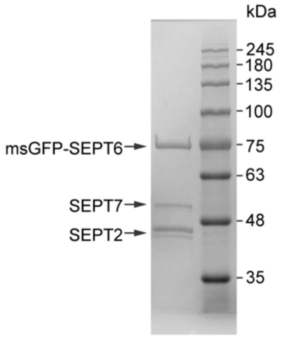

Supplementary Fig. 1 related to Fig. 1. Purification of the recombinant septin complex SEPT2-msGFP-SEPT6-SEPT7. Coomassie blue staining showing the purification of septins. Representative image from a purification repeated more than 3 independent times.

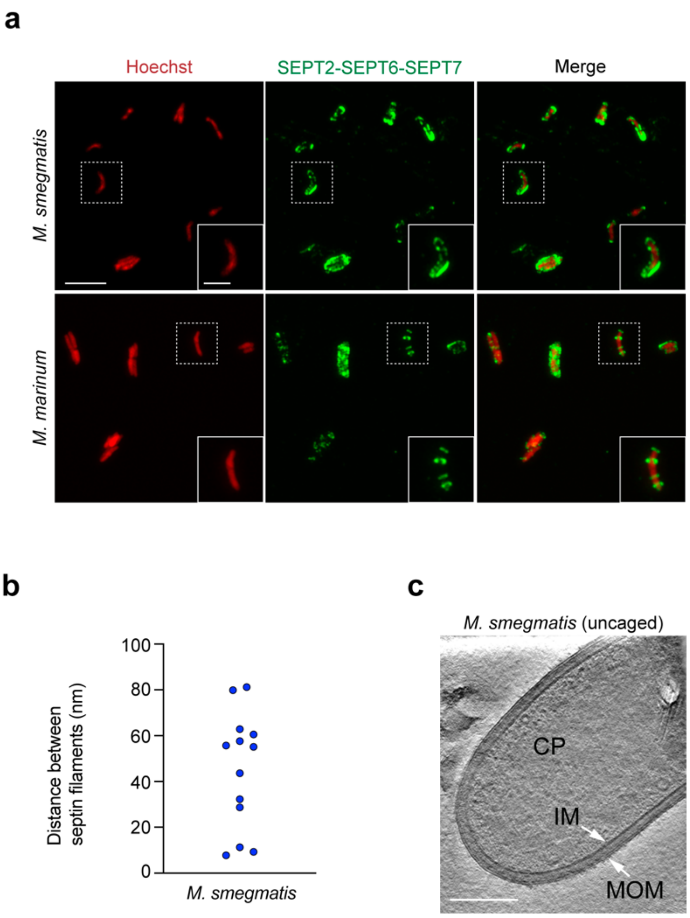

Supplementary Fig. 2 related to Fig. 1. a, Airyscan confocal images showing the binding of septins in vitro to *M. smegmatis* (top) or *M. marinum* (bottom). Scale bar, 5 µm (inset, 2 µm). **b,** Measured distance between septin filaments on the surface of *M. smegmatis*. Data corresponds to n = 13 septin filaments from 3 independent tomograms. **c,** Control cryo-ET image of non-caged *M. smegmatis* (accession No. #EMD-12565). Image shown correspond to a slice of 11 nm thickness. CP: cytoplasm; IM: inner membrane; MOM: mycobacterial outer membrane. Scale bar, 200 nm. This experiment was performed 3 independent times.

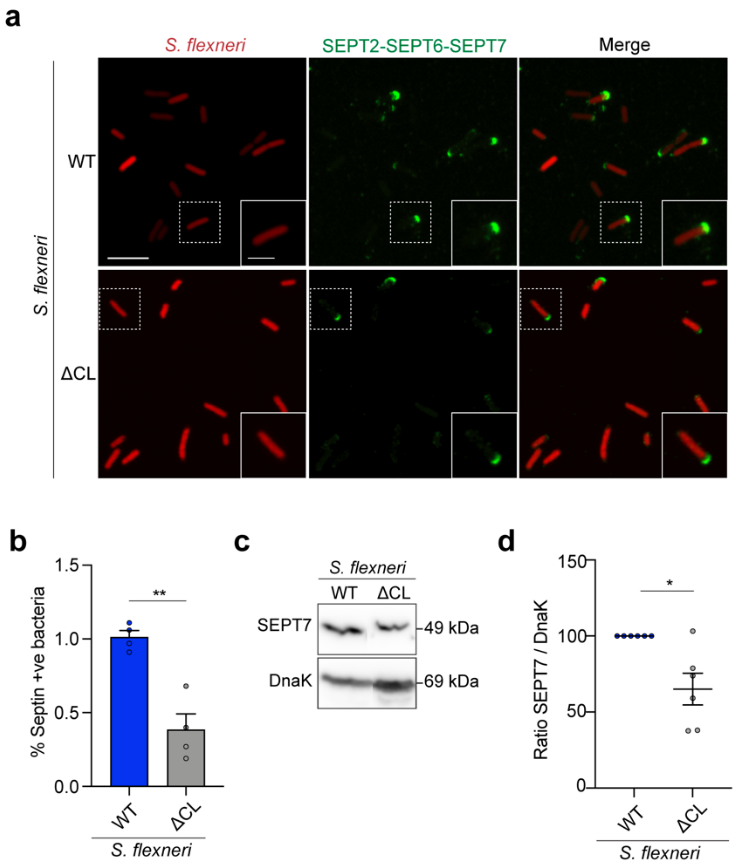

Supplementary Fig. 3 related to Fig. 2. Cardiolipin is important for *S. flexneri* recognition by septins in vitro. a, Airyscan confocal images showing the binding of septins in vitro to *S. flexneri* WT (top) or ΔCL (lacking cardiolipin). Scale bar, 5 μm (inset, 2 μm). **b,** Percentage of bacteria recruiting septins in vitro (normalized to *S. flexneri* WT mean value). M90T values also used in Fig. 2b. Data represents the mean ± SEM from n = 1,174 (WT) and n = 907 (ΔCL) *S. flexneri* cells distributed in 4 independent experiments. **, p = 0.0017 by two-tailed Student´s t-test. **c,** Representative blot of bacterial sedimentation assays performed after in vitro reconstitution assays. DnaK was used as loading control. Representative image from 4 independent experiments. **d,** Quantification of bacterial sedimentation assays from (**c**). Graphs represent the mean ± SEM of the ratio SEPT7 / DnaK from 6 independent blots. *, p = 0.048 by two-tailed Mann-Whitney´s test.

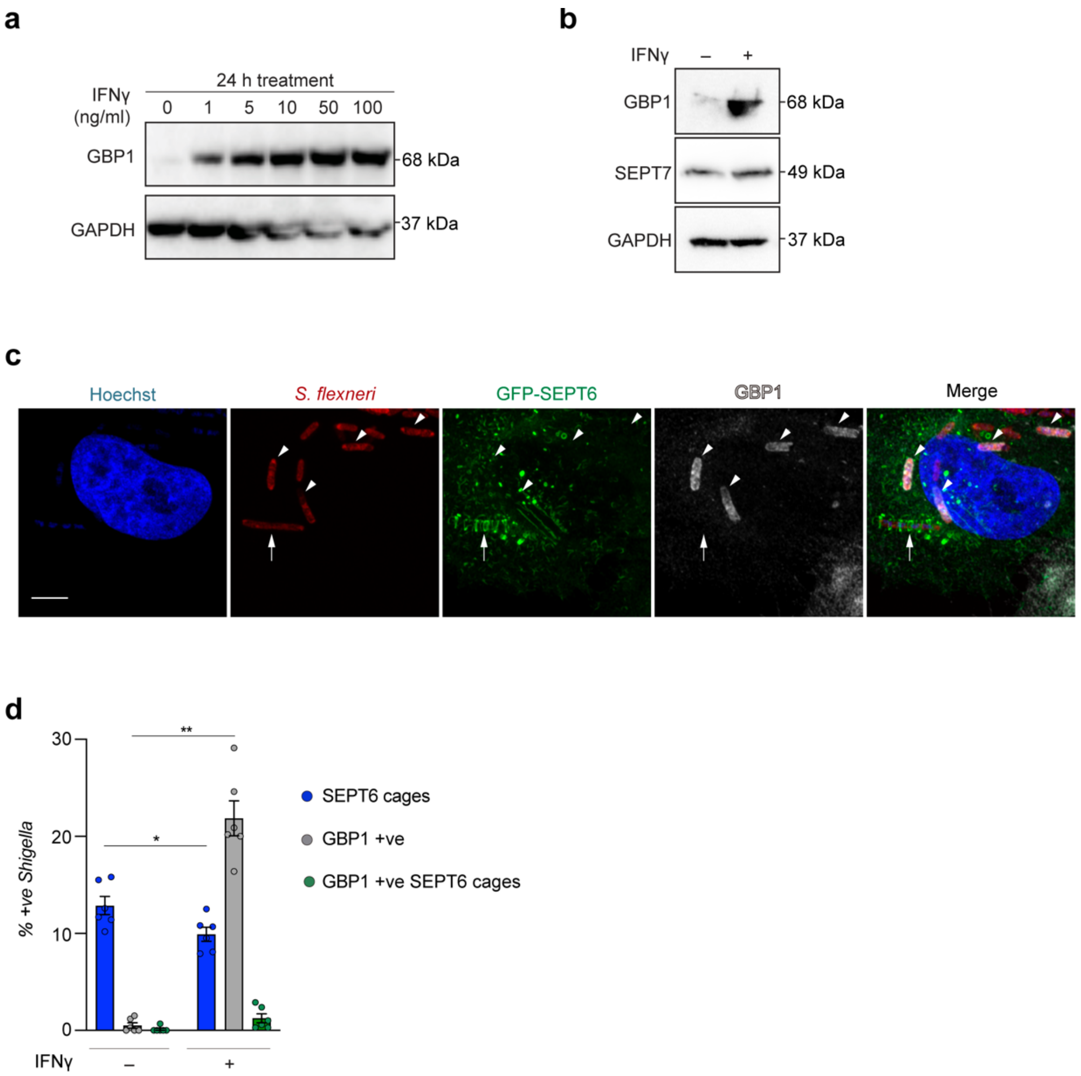

Supplementary Fig. 4 related to Fig. 2. Septins and GBPs play complementary roles during cell-autonomous immunity to *S. flexneri* infection. a, Western blot showing GBP1 production upon IFNγ stimulation in HeLa cells. GAPDH was used as loading control. Blot representative from 2 independent experiments. **b,** Western blot showing increased production of GBP1 but not SEPT7 upon stimulation of HeLa cells with 100 ng/ml of IFNγ. GAPDH was used as loading control. Representative blot from 2 independent experiments. **c,** Airyscan confocal images showing septin caged and GBP-recruiting *S. flexneri* in GFP-SEPT6-producing HeLa cells treated with 100 ng/ml of IFNγ-treated for 24 h. Arrow, septin caged *S. flexneri*; arrow heads, GBP1-recruiting *S. flexneri*. Scale bar, 5 μm. **d,** Percentage of septin caged, GBP-recruiting bacteria and double positive *S. flexneri* quantified from data collected on panel (**c**). Cells were treated or not with 100 ng/ml of IFNγ-treated for 24 h and infected with *S. flexneri* for 4 h 40 min. Data represents the mean ± SEM from n = 1,958 (non-treated) and n = 2,006 (IFNγ-treated) bacterial cells distributed in 6 independent experiments. *, p = 0.0316, by two-tailed Student´s t-test, **, p = 0.0022 by two-tailed Mann-Whitney´s test.

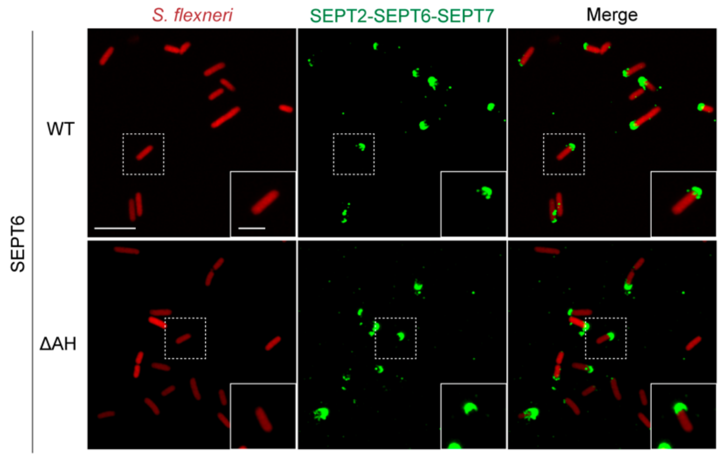

Supplementary Fig. 5 related to Fig. 3. Role of the amphipathic helix domain of human SEPT6 in curvature recognition in *S. flexneri*. a, Airyscan confocal images showing the binding in vitro of SEPT6WT- (top) or SEPT6ΔAH-containing septins complexes to *S. flexneri* WT. This experiment was performed 9 independent times. Scale bar, 5 μm (inset, 2 μm).

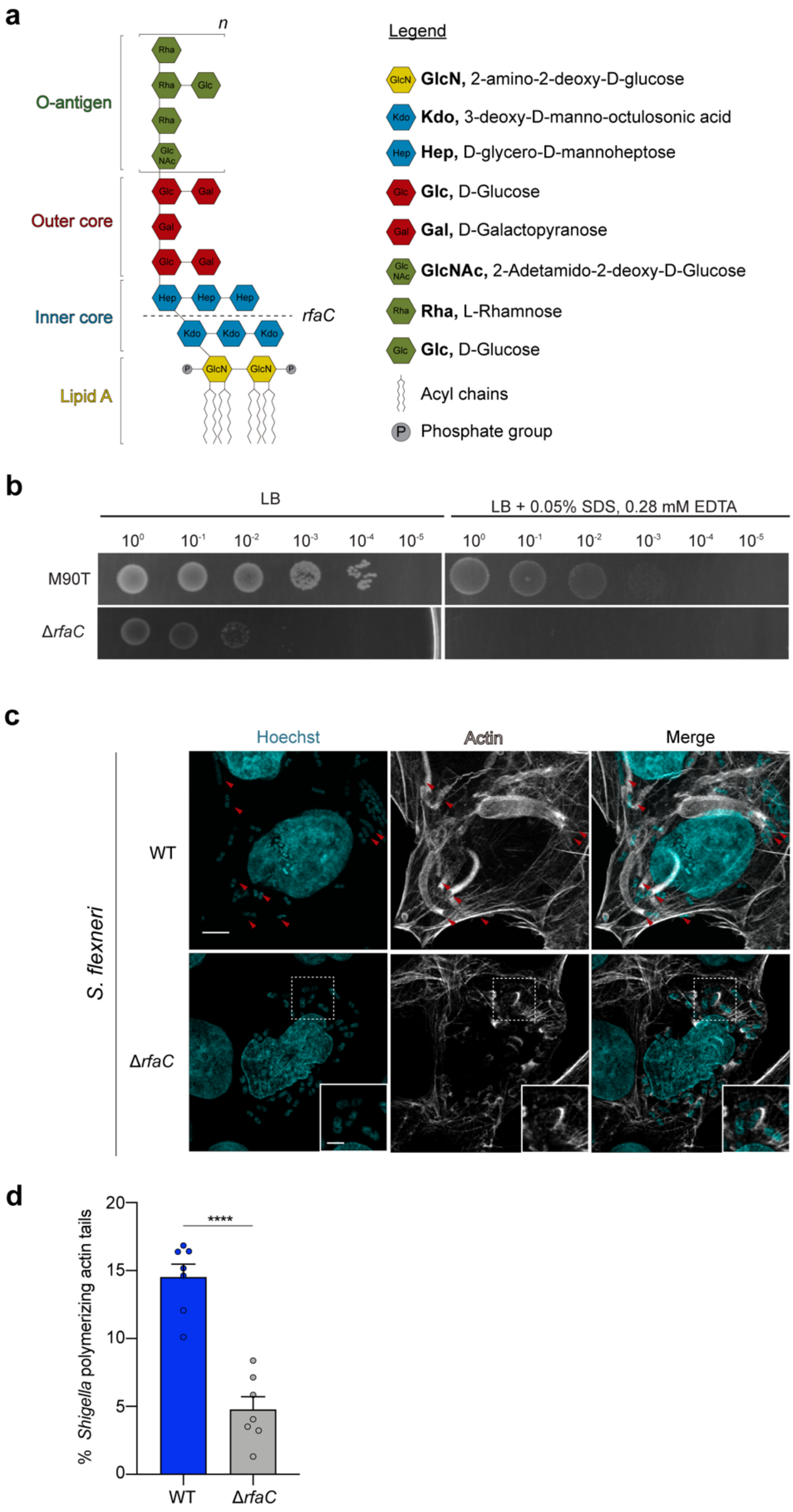

Supplementary Fig. 6 related to Fig. 4. Lack of O-antigen, inner and outer cores of the LPS increases sensitivity of *S. flexneri* to cationic detergents and decreases actin tail formation. **a,** Graphical representation of bacterial LPS of *S. flexneri* 5a. Dashed line indicates the truncation of the O-antigen in Δ*rfaC* mutant[1]. Scheme adapted from[2,3]. **b,** SDS/EDTA sensitivity assays.

Images are representative from 3 independent experiments. **c,** Airyscan confocal images of HeLa cells infected for 3 h 40 min with *S. flexneri afaI* (WT) or *S. flexneri* Δ*rfaC* and stained for Hoechst and actin (phalloidin). Bacteria polymerizing actin tails are indicated with red arrow heads. Scale bar, 5 μm (inset, 2 μm.). **d,** Percentage of bacteria polymerizing actin tails. Data represents the mean ± SEM from n = 1,435 (*afaI*) and n = 1,360 (Δ*rfaC*) *Shigella* cells distributed in 7 independent experiments. ****, $p < 0.0001$ by two tailed Student's t-test.

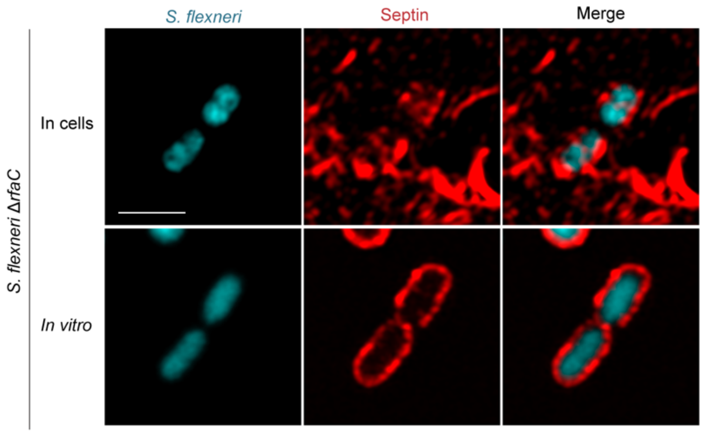

Supplementary Fig. 7 related to Fig. 4. In vitro reconstituted septin cages resemble septin cages observed during HeLa cell infection. Airyscan confocal images showing septin caged *S. flexneri ΔrfaC* during infection (top panel) or in vitro (bottom panel). Representative images from 8 independent experiments. Scale bar, 2 μm.

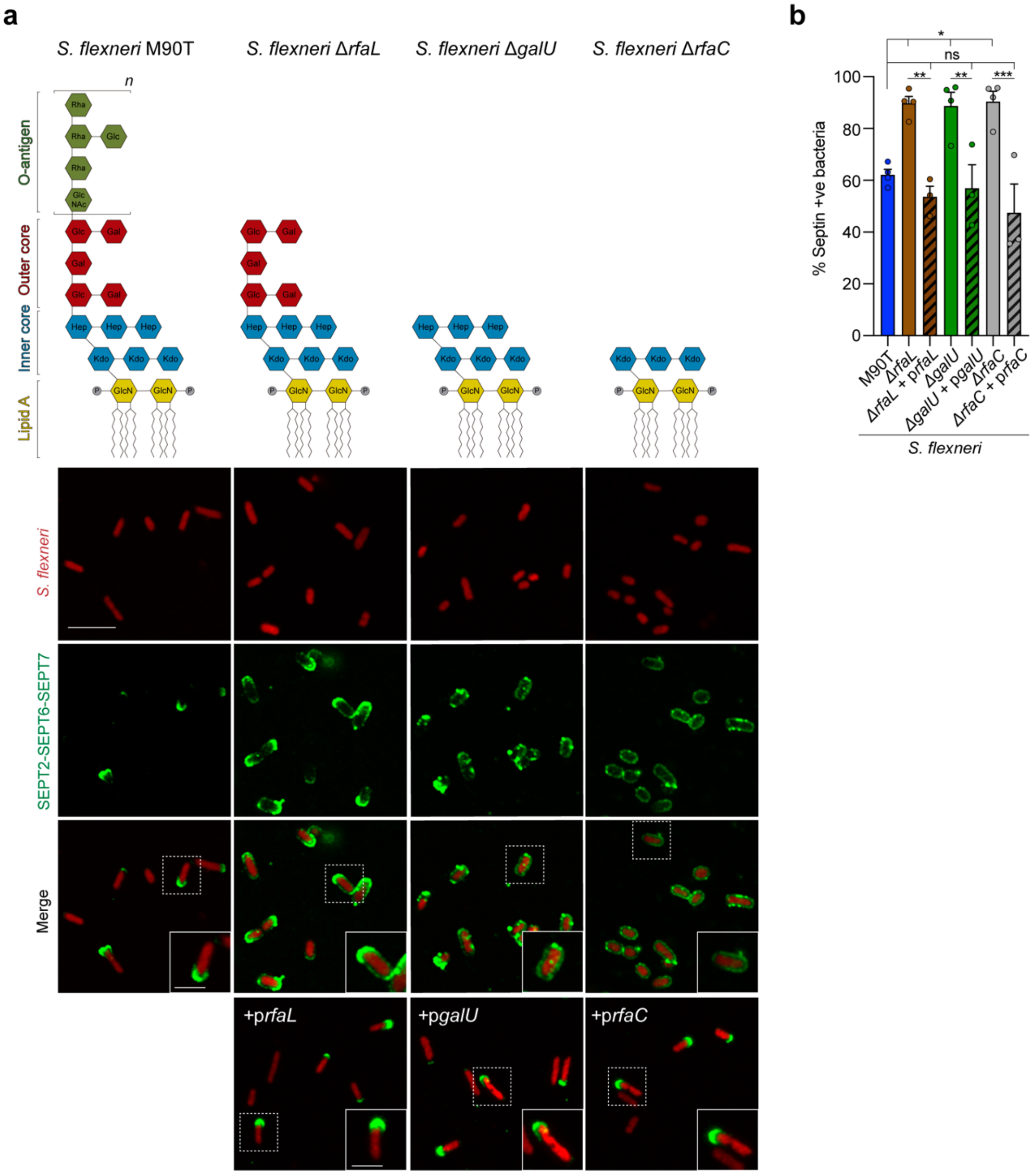

Supplementary Fig. 8 related to Fig. 4. *S. flexneri* LPS act as a physical barrier that prevents outer membrane recognition by septins. a, Schemes representing the composition of LPS and the corresponding Airyscan confocal images comparing septin binding to (from left to right) *S. flexneri* WT, *S. flexneri ΔrfaC, S. flexneri ΔrfaL* and *S. flexneri ΔgalU*. Bottom panels show complemented strain producing RfaL, GalU and RfaC. Scale bar, 5 μm (inset 2 μm). **b,** Percentage of bacteria recruiting septins in vitro. Quantifications represent mean ± SEM from n = 598 (WT), n = 1,150 (Δ*rfaC*), n = 716 (Δ*rfaC*+*prfaC*), n = 1,075 (Δ*rfaL*), n = 463 (Δ*rfaL*+*prfaL*), n = 1,109 (Δ*galU*), and n = 499 (Δ*galU*+*pgalU*) *S. flexneri* cells distributed in at least 3 independent experiments. ns, non-significant, * $p < 0.05$, **, $p < 0.01$, ***, $p = 0.0007$ by by one-way ANOVA and Tukey's post-test.

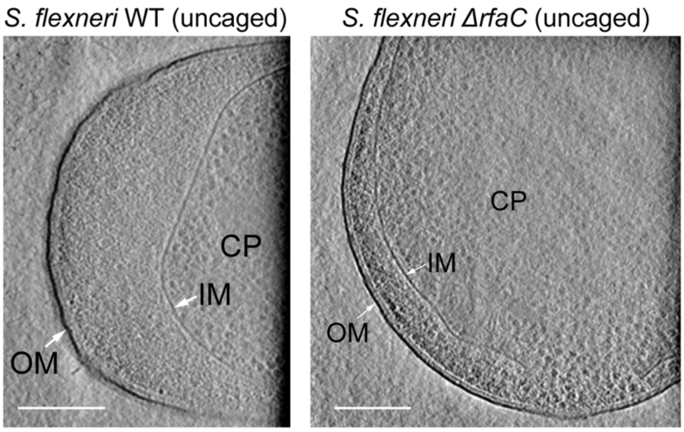

Supplementary Fig. 9 related to Fig. 6d. Control cryo-ET images of non-caged *S. flexneri*. *S. flexneri* WT (left panel, accession No. #EMD-12578) or *S. flexneri* Δ*rfaC* (right panel, accession No. #EMD-12580) were cultured in the absence of septin complexes and imaged by cryo-ET. In this case no structures corresponding to septins were observed. Images shown correspond to slices of 10.8 nm thickness. CP: cytoplasm; IM: inner membrane; OM: outer membrane. Scale bar, 200 nm. This experiment was performed 3 independent times.

Supplementary Table 1. List of bacterial strains and plasmids used in this study.

Strain or plasmid	Genotype	Reference
Shigella flexneri srv. 5a str. M90T	Sm^R	[4]
S. flexneri Δ*mxiD*	T3SS⁻, Kan^R, Sm^R	[4]
S. flexneri ΔicsB	*icsB*⁻, Kan^R, Sm^R	[4]
S. flexneri Δ*icsA*	*icsA*⁻, Sm^R	[4]
S. flexneri afaI	Constitutively producing the adhesin AfaE, $Carb^R$, Sm^R	[4]
S. flexneri Δ*icsA* picsA	*icsA*⁻, Plasmid encoding and constitutively expressing *icsA*, $Carb^R$, Sm^R	This study
S. flexneri Δ*rfaC*	*rfaC*⁻, $kanamycin^R$, Sm^R	This study
S. flexneri Δ*rfaL*	*rfaL*⁻, $kanamycin^R$, Sm^R	This study
S. flexneri Δ*galU*	*galU*⁻, $kanamycin^R$, Sm^R	This study
S. flexneri Δ*rfaC*Δ*icsA*	*rfaC*⁻, icsA⁻, $kanamycin^R$, Sm^R	This study
S. flexneri Δ*rfaL*Δ*icsA*	*rfaL*⁻, icsA⁻, $kanamycin^R$, Sm^R	This study
S. flexneri Δ*galU*Δ*icsA*	*galU*⁻, icsA⁻, $kanamycin^R$, Sm^R	This study
S. flexneri ΔCL	Δ*cls*Δ*ymdC*Δ*ybhO,* Kan^R	[5]
S. flexneri mCherry	Constitutively producing mCherry, $Carb^R$, Sm^R	[6]
S. flexneri Δ*rfaL* mCherry	Constitutively producing mCherry, $Carb^R$, Sm^R	This study
Escherichia coli DH5α	F⁻ φ80*lac*ZΔM15 Δ(*lac*ZYA-*arg*F) U169 *rec*A1 *end*A1 *hsd*R17(r_K^-, m_K^+) *pho*A *sup*E44 λ⁻ *thi*-1 *gyr*A96 *rel*A1	ThermoScientific (#18265017)
E. coli BL21 (DE3)	F⁻ *ompT gal dcm lon hsdSB* (*rB⁻mB⁻*) pLysS, Cm^R	ThermoScientific (#EC0114)
E. coli BL21 (DE3) picsA	Plasmid encoding and constitutively expressing *icsA*, $Carb^R$	This study
Mycobacterium marinum	$Hygromycin^R$	[4]
Mycobacterium smegmatis mc^2155		(Gift from Gerald Larrouy-Maumus lab)
Mycobacterium smegmatis DsRed	*dsRed*	(Gift from Haig Alexander Eskandarian)
**Plasmids**		
pKD46	*repA101*(ts) *oriR101* bla *ParaB*-l-Red recombinase, $Carb^R$	[7]
pKD4	$oriR_{\gamma R6k}$ *FRT::kan::FRT*, Kan^R	[7]
pCP20		[7]
$picsA^{507\text{-}620}$-mCherry	P_{lac}, IcsA derivate containing polar localization sequence	[8]
pnEA-vH-SEPT2	P^{lac}-*SETP2*	[9]
pnCS-SEPT6-SEPT7	P_{lac}-*SEPT6-SETP7*	[9]
pnCS-msGFP–SEPT6-SEPT7	P_{lac}-*msGFP–SEPT6-SEPT7*	This study
pnCS-msGFP–SEPT6ΔAH-SEPT7	P^{l}_{ac}-*msGFP–SEPT6*ΔAH-*SEPT7*	This study
pLVX	$P_{CMV\ IE}$, $Puro^R$, $Carb^R$	Takara Bio Inc.
psPAX2	*gag, pol, rev, tat*, $Carb^R$	Trono lab 2nd-generation packaging system; Addgene
pMD2.g	VSV-G envelope, $Carb^R$	Trono lab 2nd-generation packaging system; Addgene
pLVX-msGFP–SEPT6	$P_{CMV\ IE}$-*msGFP–SEPT6*, $Puro^R$, $Carb^R$	This study
pLVX-msGFP–SEPT6ΔAH	$P_{CMV\ IE}$-*msGFP–SEPT6ΔAH*, $Puro^R$, $Carb^R$	This study
pFUS-PBAD	P_{BAD} promoter, $lacI^q$, Kan^R	[10]
prfaC	P_{BAD}-rfaC, $lacI^q$, Kan^R	This study
prfaL	P_{BAD}-rfaL, $lacI^q$, Kan^R	This study
pgalU	P_{BAD}-galU, $lacI^q$, Kan^R	This study

Supplementary Table 2. List of primers used in this study.

Primer	Sequence (5´-3´)	Use
Fw-del-rfaC	CACTGATGCCCAGCAGGCAATCCCAGGGATTAAGTTTGACTGGGTGGTGGGT GTAGGCTGGAGCTGCTTC	Deletion of *rfaC*
Rv-del-rfaC	AAGAGACATACTTGTAGAACGACACTCTACTTGATTCTTCCCATACCCACATGG GAATTAGCCATGGTCC	Deletion of *rfaC*
rfaC-Comp5	GACTTCACACCGCCGCTATCCCATAAAG	Confirmation of *rfaC* deletion
rfaC-Comp3	GAGAGATAGTGTTTTGGCCGTTTGCAGAG	Confirmation of *rfaC* deletion
Fw-del-rfaL	GCGTACTGGAACAGAGCTCTCGTATTCTTATTCATTACCACCTATTTTTTGGTG TAGGCTGGAGCTGCTTC	Deletion of *rfaL*
Rv-del-rfaL	GCCGGCGTAAACGCCTAATAAATTTGGTTCAATTTGTTCTACGTTTCCACGGAC ATGGGAATTAGCCATGGTCC	Deletion of *rfaL*
rfaL-Comp5	GGAAGAATCAAGTAGAGTGTCGTTCTACAAGTATG	Confirmation of *rfaL* deletion
rfaL-Comp3	GGGATGGCGTAACTCAAAGATTCGAAGAG	Confirmation of *rfaL* deletion
Fw-del-galU	CGGCGTCGATTGCTCAACGCCGTTTCGTGGATAACACCGATACGGATGTTAGT GTAGGCTGGAGCTGCTTC	Deletion of *galU*
Rv-del- galU	CGCCATTCGTATAAGTAATTGTCTTAATTATGCTATCTCGCCTCCTTTTCAGACA TGGGAATTAGCCATGGTCC	Deletion of *galU*
galU-Comp5	GCCAGCGGCGGGATTTTTTATTGTC	Confirmation of *galU* deletion
galU-Comp3	CCAGGTGCAAATCAGTAATGGTGTTCC	Confirmation of *galU* deletion
Fw-del-icsA	CCCGTTGCATTGATATATAACACAGCTCTCATGTTTTGGTTGAGGCTTTGTTTG TGTAGGCTGGAGCTGCTTC	Deletion of *icsA*
Rv-del-icsA	CCAGTTTTGAGTTCAATCACATTACGGTTGCCTATCTGGTGATTTGACATGCGA CATGGGAATTAGCCATGGTCC	Deletion of *icsA*
icsA-Comp5	CCCCTCTTTTTTTCAAAGCAAGACACAGG	Confirmation of *icsA* deletion
icsA-Comp3	CCCACACCAGTTTTGAGTTCAATCACATTAC	Confirmation of *icsA* deletion
pKD4-Comp5	GCATCGCCTTCTATCGCCTTCTTG	Confirmation of *rfaC* deletion
pKD4-Comp3	CCTGCGTGCAATCCATCTTGTTCA	Confirmation of *rfaC* deletion
Spe-SD-GFP-5	CCCCACTAGTAATAATTTTGTTTAACTTTAAGAAGGAGATATACATATGAGTAAA GGTGAAGAACTGTTCACCGGTGTTGTTC	Amplification/cloning *msGFP*
TEV-Xba-GFP-3	CCCGGAGCCTTGGAAGTAGAGGTTCTCTCTAGACCCGCCTTTGTAGAGTTCAT CCATGCCGTGCGTG	Amplification/cloning *msGFP*
Xba-TEV-S6-5	TACAAAGGCGGGTCTAGAGAGAACCTCTACTTCCAAGGCTCCGGGGCAGCGA CCGATATAGCTCGCCAG	Amplification/cloning *SEPT6*
Pst-Cla-S6	GGCCTGCAGGTAGGCCTCGAATTGTGCATCGATG	Amplification/cloning *SEPT6*
GFP-S6-seq-5	CCGGGATCTCGACGCTCTCCCTTATG	*msGFP* sequencing
GFP-S6-seq-3	GGGGGACAGTTCGGCAACCTTCAC	*msGFP-SEPT6* sequencing
SEPT6-seq-inter5	GGACAGCTACAAGCCTATCGTG	*SEPT6* sequencing
SEPT6 sec 5'	GGCCTACCTGCAGGAAGAGCTAAAGATC	*SEPT6* sequencing
SEPT6 sec 3'	CGGGAGTCATGGTAGGTGTGTAGCAC	*SEPT6* sequencing
pLVX_fwd	TCCCGCGACTCTAGATAATTC	Vector amplification for Gibson assembly
pLVX_rv	GGTCGGTGTCTTCTATGG	Vector amplification for Gibson assembly
msGFP-SEPT6_fwd	CTCCATAGAAGACACCGACCATGAGTAAAGGTGAAGAAC	*msGFP–SEPT6* amplification for Gibson assembly
msGFP-SEPT6_rv	AATTATCTAGAGTCGCGGGATTAATTTTTCTTCTCTTTGTCTC	*msGFP–SEPT6* amplification for Gibson assembly
msGFP-N-SEPT6-fwd	GAAAAATTAAATGAGTAAAGGTGAAGAAC	*SEPT6ΔAH* N-terminus amplification for Gibson assembly
msGFP-N-SEPT6-rv	CGTCCTGGTGCTCCGCTTCTTTCTCTTTG	*SEPT6ΔAH* N-terminus amplification for Gibson assembly
C-SEPT6-fwd	AGAAGCGGAGCACCAGGACGAGAAGAAG	*SEPT6ΔAH* C-terminus amplification for Gibson assembly
C-SEPT6-rv	CTTTACTCATTTAATTTTTCTTCTCTTTGTCTCTC	*SEPT6ΔAH* C-terminus amplification for Gibson assembly
pLVX-sec	GGGGCTGCTAAAGCGCATGC	Sequencing
SEPT6-seq-inter5.2	GGCCAAAAGGAACGAGTTCCTAG	Sequencing
pnCS-fwd	GGATCCTAATAGTCTAGTAATAATTTTG	Vector amplification for Gibson assembly
pnCS-rv	ATGTATATCTCCTTCTTAAAGTTAAAC	Vector amplification for Gibson assembly
msGFP-S6ΔAH_fwd	TTTAAGAAGGAGATATACATATGAGTAAAGGTGAAGAAC	*msGFP– SEPT6ΔAH* amplification for Gibson assembly
msGFP-S6ΔAH_rv	TTACTAGACTATTAGGATCCTTAATTTTTCTTCTCTTTGTCTC	*msGFP– SEPT6ΔAH* amplification for Gibson assembly
pFUS-Gib-fw	ACTAGTGGTACATGTCGTC	Amplification for Gibson assembly
pFUS-Gib-rv	GACGTCTTCCTCCCTTTAC	Amplification for Gibson assembly
rfaC-fw	CGTAAAGGGAGGAAGACGTCATGCGGGTTTTGATCGTTAAAAC	Amplification for Gibson assembly
rfaC-rv	CGACGACATGTACCACTAGTTCATCTTATCTCCGATGTCAAC	Amplification for Gibson assembly
rfaL-fw	CGTAAAGGGAGGAAGACGTCATGACCTCAACATTATTTTTCTC	Amplification for Gibson assembly
rfaL-rv	CGACGACATGTACCACTAGTTTACTTGTTTTTCATCGCTAATAATAAG	Amplification for Gibson assembly
galU-fw	CGTAAAGGGAGGAAGACGTCATGGCTGCCATTAATACG	Amplification for Gibson assembly
galU-rv	CGACGACATGTACCACTAGTTTACTTCTTAATGCCCATC	Amplification for Gibson assembly

Supplementary references

1 Xu, D., Zhang, W., Zhang, B., Liao, C. & Shao, Y. Characterization of a biofilm-forming *Shigella flexneri* phenotype due to deficiency in Hep biosynthesis. *PeerJ* **4**, e2178, doi:10.7717/peerj.2178 (2016).

2 Anderson, M., Sansonetti, P. J. & Marteyn, B. S. *Shigella* Diversity and Changing Landscape: Insights for the Twenty-First Century. *Front Cell Infect Microbiol* **6**, 45, doi:10.3389/fcimb.2016.00045 (2016).

3 Kutsch, M. *et al.* Direct binding of polymeric GBP1 to LPS disrupts bacterial cell envelope functions. *EMBO J* **39**, e104926, doi:10.15252/embj.2020104926 (2020).

4 Mostowy, S. *et al.* Entrapment of intracytosolic bacteria by septin cage-like structures. *Cell Host Microbe* **8**, 433-444, doi:10.1016/j.chom.2010.10.009 (2010).

5 Krokowski, S. *et al.* Septins Recognize and Entrap Dividing Bacterial Cells for Delivery to Lysosomes. *Cell Host Microbe* **24**, 866-874 e864, doi:10.1016/j.chom.2018.11.005 (2018).

6 Mostowy, S. *et al.* The zebrafish as a new model for the in vivo study of *Shigella flexneri* interaction with phagocytes and bacterial autophagy. *PLoS Pathog* **9**, e1003588, doi:10.1371/journal.ppat.1003588 (2013).

7 Datsenko, K. A. & Wanner, B. L. One-step inactivation of chromosomal genes in *Escherichia coli* K-12 using PCR products. *Proc Natl Acad Sci U S A* **97**, 6640-6645, doi:10.1073/pnas.120163297 (2000).

8 Nilsen, T., Yan, A. W., Gale, G. & Goldberg, M. B. Presence of multiple sites containing polar material in spherical *Escherichia coli* cells that lack MreB. *J Bacteriol* **187**, 6187-6196, doi:10.1128/JB.187.17.6187-6196.2005 (2005).

9 Mavrakis, M. *et al.* Septins promote F-actin ring formation by crosslinking actin filaments into curved bundles. *Nat Cell Biol* **16**, 322-334, doi:10.1038/ncb2921 (2014).

10 Lobato-Marquez, D., Moreno-Cordoba, I., Figueroa, V., Diaz-Orejas, R. & Garcia-del Portillo, F. Distinct type I and type II toxin-antitoxin modules control *Salmonella* lifestyle inside eukaryotic cells. *Sci Rep* **5**, 9374, doi:10.1038/srep09374 (2015).
