# Supplementary material for: Mechanistic insight into bacterial entrapment by septin cage reconstitution
Source: Nat Commun. 2021 Jul 23;12:4511. doi: 10.1038/s41467-021-24721-5 (PMC8302635; doi:10.1038/s41467-021-24721-5)
Supplement: Supplementary file 3 — Description of Additional Supplementary Files [file 41467_2021_24721_MOESM3_ESM.pdf]

Description of Additional Supplementary Files

File Name: Supplementary Movie 1
Description: Septin cage entrapment of *S. flexneri ΔrfaL* (related to Fig. 5b). Time-lapse epifluorescence microscopy showing the recruitment of SEPT6WT-containing septin complexes (green) to *S. flexneri ΔrfaL* mCherry. Time is shown as min:s (top right corner). Scale bar, 2 µm.

File Name: Supplementary Movie 2
Description: Septin cage entrapment of *M. smegmatis* (related to Fig. 5b). Time-lapse epifluorescence microscopy showing the recruitment of SEPT6WT-containing septin complexes (green) to *M. smegmatis* DsRed. Time is shown as min:s (top right corner). Scale bar, 2 µm.

File Name: Supplementary Movie 3
Description: *E. coli* is not entrapped in septin cages (related to Fig. 5). Time-lapse epifluorescence microscopy showing the absence of recruitment of SEPT6WT-containing septin complexes (green) to *E. coli*. Time is shown as min:s (top right corner). Scale bar, 5 µm.

File Name: Supplementary Movie 4
Description: Septin cage entrapment of *S. flexneri ΔrfaL* (related to Fig. 5b). Time-lapse epifluorescence microscopy showing the recruitment of SEPT6DAH-containing septin complexes (green) to *S. flexneri ΔrfaL* mCherry. Time is shown as min:s (top right corner). Scale bar, 2 µm.

File Name: Supplementary Software
Description: A custom phyton script (Supplementary Software related to Fig. 1h and 6e) was developed to quantify the distance between septin filaments and the bacterial surface. A custom MATLAB script (Supplementary Software related to Fig. 5) was developed for image analysis and rate calculation.
