# Supplementary material for: Mechanistic insight into bacterial entrapment by septin cage reconstitution
Source: Nat Commun. 2021 Jul 23;12:4511. doi: 10.1038/s41467-021-24721-5 (PMC8302635; doi:10.1038/s41467-021-24721-5)
Supplement: Supplementary file 8 — Supplementary Software [file 41467_2021_24721_MOESM8_ESM.zip › Supplementary File 2 related to Fig. 5.docx]

% load cell contours from Morphometrics (frame and BW)

% load GFP images (SepImgAll)

count = 1;

for i = 1:size(SepImgAll,1)

clear v1 v2 v bg x

v1 = squeeze(SepImgAll(i,:,:)); %GFP image at ith time point

vm = median(v1(:));

BWt = bwmorph(BW,'thicken',2); %cell mask

x = ~BWt .* v1; %GFP image outside of the mask

x(x==0) = nan;

bgAll(count,:) = nanmedian(x(:));

interval = 1; %in pixels

dinterval = 0.5;

[Vq{count},localBG{count}] = GetIntensityatContour(frame,v1,interval, dinterval,x);

count = count+1;

end

localBG = cell2mat(localBG');

%%

localBG = localBG./max(localBG);

dBG = diff(localBG);

[a,maxID] = max(dBG); %time point when septins arrive at the fov

x = 1:size(dBG,1);

for k = 1:size(Vq{1},2)

clear x

for i = 1:size(Vq,2)

x(i,:) = Vq{i}{k};

end

if(sum(isnan(x(:)))==0)

IperCell{k} = x;

end

end

IperCell(cellfun(@isempty,IperCell))=[];

%%

th = eps; %to detect when signal becomes higher than background

for k = 1:size(IperCell,2)

clear x y y0 a b c ind

x = IperCell{k}(maxID(k):end,:);

y = mean(x,2);

y = y./max(y);

y0 = y'; y0(y0<0) = nan;

ind = interp1(y,1:length(y),th,'nearest');

b = ind;

t0(k,:) = [maxID(k) b];

end

%%

g = fittype('a-b*exp(-c*x)');

dt = 10; %in sec

t = [1:size(Vq,2)]' .*dt ./60;

for k = 1:size(IperCell,2)

clear x y f0 gof y0 a b t1

x = IperCell{k}(sum(t0(k,:)):end,:);

t1 = t(sum(t0(k,:)):end);

y = mean(x,2);

y = y./max(y);

[f0,gof] = fit(t1,y,g,'StartPoint',[[ones(size(t1)), -exp(-t1)]\y; 1]);

xx = linspace(min(t1),max(t1),10);

gofVals(k) = gof.rsquare;

figure,hold on,

plot(t1,y,'o-','linewid',1.2)

plot(xx,f0(xx),'r-','linewid',1.2)

r(k) = f0.c;

end

%%%%%%%%%%%%%%%%%%%%%%%%%%%%%%%%%%%%%%%%%%%%%%%%%%%%%%%%%%%%%%%%%%%%%%%%

function [Vq,localBG] = GetIntensityatContour(frame,v1,interval, dinterval,BG)

for k = 1:size(frame.object,2)

clear bbx bby *xy* dx* dy* slope1 P3 a

bbx = frame.object(k).Xcont;

bby = frame.object(k).Ycont;

xy = [bbx;bby]';

xy = [xy;[xy(1,:)]];

[xy,~] = MakeContour_EquiDistantPts(xy); %0.5 pixel spacing

xy(end,:) = [];

%%% find coordinates of the points to interpolate intensities

dy = gradient(xy(:,2));

dx = gradient(xy(:,1));

dy_N = dy ./ sqrt(dx.^2 + dy.^2);

dx_N = dx ./ sqrt(dx.^2 + dy.^2);

slope1 = [-dy_N , dx_N];

Xq_P3 = []; Yq_P3 = [];

dummy=1;

for d = -interval:dinterval:interval

P3 = xy - slope1 * d;

Xq_P3(:,dummy) = P3(:,1);

Yq_P3(:,dummy) = P3(:,2);

dummy=dummy+1;

end

a = interp2(v1,Xq_P3,Yq_P3); %interpolate image v1 at [Xq_P3,Yq_P3]

if(numel(BG)>2) %find local background

b = round(mean(xy))-30;

rect = imcrop(BG,[b(1) b(2) 75 75]);

localBG(k) = nanmedian(rect(:));

Vq{k} = mean(a,2) - localBG(k); %signal after local BG subtraction

end

end

%%%%%%%%%%%%%%%%%%%%%%%%%%%%%%%%%%%%%%%%%%%%%%%%%%%%%%%%%%%%%%%%%%%%%%%%

function xy1 = MakeContour_EquiDistant(P1)

d = (sqrt(sum(diff(P1).^2,2)));

l = sum(sqrt(sum(diff(P1).^2,2)));

n1 = round(l/0.5);

xy1 = curvspace(P1,n1);
