# Supplementary material for: Mechanistic insight into bacterial entrapment by septin cage reconstitution
Source: Nat Commun. 2021 Jul 23;12:4511. doi: 10.1038/s41467-021-24721-5 (PMC8302635; doi:10.1038/s41467-021-24721-5)
Supplement: Supplementary file 9 — Reporting Summary [file 41467_2021_24721_MOESM9_ESM.pdf]

nature research

Corresponding author(s): Damián Lobato-Márquez
Serge Mostowy

Last updated by author(s): Jun 16, 2021

Reporting Summary

Nature Research wishes to improve the reproducibility of the work that we publish. This form provides structure for consistency and transparency in reporting. For further information on Nature Research policies, see our Editorial Policies and the Editorial Policy Checklist.

Statistics

For all statistical analyses, confirm that the following items are present in the figure legend, table legend, main text, or Methods section.

n/a	Confirmed	
☐	☒	The exact sample size (n) for each experimental group/condition, given as a discrete number and unit of measurement
☐	☒	A statement on whether measurements were taken from distinct samples or whether the same sample was measured repeatedly
☐	☒	The statistical test(s) used AND whether they are one- or two-sided<br>*Only common tests should be described solely by name; describe more complex techniques in the Methods section.*
☐	☒	A description of all covariates tested
☐	☒	A description of any assumptions or corrections, such as tests of normality and adjustment for multiple comparisons
☐	☒	A full description of the statistical parameters including central tendency (e.g. means) or other basic estimates (e.g. regression coefficient) AND variation (e.g. standard deviation) or associated estimates of uncertainty (e.g. confidence intervals)
☐	☒	For null hypothesis testing, the test statistic (e.g. F, t, r) with confidence intervals, effect sizes, degrees of freedom and P value noted<br>*Give P values as exact values whenever suitable.*
☒	☐	For Bayesian analysis, information on the choice of priors and Markov chain Monte Carlo settings
☒	☐	For hierarchical and complex designs, identification of the appropriate level for tests and full reporting of outcomes
☒	☐	Estimates of effect sizes (e.g. Cohen's d, Pearson's r), indicating how they were calculated

Our web collection on statistics for biologists contains articles on many of the points above.

Software and code

Policy information about availability of computer code

Data collection	Confocal images were taken on a Zeiss LSM 880 confocal microscope driven by ZEN Black software. Live imaging was performed on a AxioObserver Z1 fluorescence microscope driven by ZEN Blue 2.3 software coupled to a MERCK Cell ASICS ONIX2 microfluidic device.
Data analysis	ZenBlue (Zeiss) v3.1, ZenBlack (Zeiss) v2.3, SerialEM v3.8.0, Fiji (ImageJ) v2.1.0, Filename_Randomizer, IMOD v4.0.29, GraphPad Prism v9, Benchling, MATLAB v.2019a, Python. Phyton (Supplementary file 1 related to Fig. 1h and 6e) and MATLAB (Supplementary file 2 related to Fig. 5) scripts were deposited in Github (https://github.com/xujweth/septin_scripts).

For manuscripts utilizing custom algorithms or software that are central to the research but not yet described in published literature, software must be made available to editors and reviewers. We strongly encourage code deposition in a community repository (e.g. GitHub). See the Nature Research guidelines for submitting code & software for further information.

Data

Policy information about availability of data

All manuscripts must include a data availability statement. This statement should provide the following information, where applicable:
- Accession codes, unique identifiers, or web links for publicly available datasets
- A list of figures that have associated raw data
- A description of any restrictions on data availability

All data are included in the manuscript. Source data are provided with this paper. Materials can be obtained from the corresponding authors upon request.

Field-specific reporting

Please select the one below that is the best fit for your research. If you are not sure, read the appropriate sections before making your selection.

- [x] Life sciences
- [ ] Behavioural & social sciences
- [ ] Ecological, evolutionary & environmental sciences

For a reference copy of the document with all sections, see nature.com/documents/nr-reporting-summary-flat.pdf

Life sciences study design

All studies must disclose on these points even when the disclosure is negative.

Sample size	No statistical methods were used to predetermine sample size. Given the small experimental variation in clonal cell lines and bacterial strains, a minimum of 3 independent biological replicates (developed independent days) were used per experiment.
Data exclusions	No data was excluded from the analysis
Replication	All experiments were performed at least 3 independent times (except Fig. 5) using independent samples from different days. All attempts at replication were successful.
Randomization	We did not use any animal model/human samples in this study. We did not use any randomization; all experiments were performed using common pools of cells and bacteria.
Blinding	For counts of Fig 3i, Supplementary Fig. 4d no blinding was applied, because phenotypes were very clear revealing the sample identity. In the rest of the samples were blinding was necessary, samples were blinded using the Fiji´s Filename_Randomizer pluging

Reporting for specific materials, systems and methods

We require information from authors about some types of materials, experimental systems and methods used in many studies. Here, indicate whether each material, system or method listed is relevant to your study. If you are not sure if a list item applies to your research, read the appropriate section before selecting a response.

Materials & experimental systems

n/a	Involved in the study	
[ ]	[x]	Antibodies
[ ]	[x]	Eukaryotic cell lines
[x]	[ ]	Palaeontology and archaeology
[x]	[ ]	Animals and other organisms
[x]	[ ]	Human research participants
[x]	[ ]	Clinical data
[x]	[ ]	Dual use research of concern

Methods

n/a	Involved in the study	
[x]	[ ]	ChIP-seq
[x]	[ ]	Flow cytometry
[x]	[ ]	MRI-based neuroimaging

Antibodies

Antibodies used	Rabbit anti-SEPT7 (1:1,000, #18991, IBL), mouse anti-DnaK (1:5,000, #ADI-SPA-880, Enzo), mouse anti-GFP (1:4,000, #ab1218, abcam), rabbit anti-GBP1 (1:1,000, #15303-1-AP, Proteintech), mouse anti-GAPDH (1:1,000 or 1:2,000, #ab8245, Abcam), goat HRP-conjugated anti-mouse (1:5,000, #P0260, Dako), goat HRP-conjugated anti-rabbit (1:5,000, #P0448, Dako), Alexa-555-conjugated anti-rabbit antibody (1:500, #10082602, ThermoFisher Scientific), Alexa-647-conjugated anti-rabbit antibody (1:500, #A27040, ThermoFisher Scientific)
Validation	All antibodies used in this study have been used in previous publications (Mostowy et al., Cell Host Microbe, 2010; Sirianni et al., EMBO Rep, 2016; Krokowski et al., Cell Host Microbe, 2018; Wandel et al., Cell Host Microbe, 2017). ThermoScientific has also validated secondary antibodies.

Eukaryotic cell lines

Policy information about cell lines

Cell line source(s)	HeLa cells were obtained from ATCC. msGFP-SEPT6 producing cells were designed in house using as template ATCC´s HeLas. HEK293FT cells were a kind gift from Michael Way.
Authentication	We obtained HeLa cells from ATCC. The cell lines were not authenticated.
Mycoplasma contamination	Cell lines were tested for Mycoplasma infection and tested negative.

Commonly misidentified lines (See ICLAC register)	No commonly misidentified cell lines were used in the study.
